# The *gp63* Gene Cluster Is Highly Polymorphic in Natural *Leishmania* (*Viannia*) *braziliensis* Populations, but Functional Sites Are Conserved

**DOI:** 10.1371/journal.pone.0163284

**Published:** 2016-09-20

**Authors:** Lilian S. Medina, Bruno Araújo Souza, Adriano Queiroz, Luiz Henrique Guimarães, Paulo Roberto Lima Machado, Edgar M Carvalho, Mary Edythe Wilson, Albert Schriefer

**Affiliations:** 1 Serviço de Imunologia, Hospital Universitário Professor Edgard Santos, Universidade Federal da Bahia, Salvador, Bahia, Brazil; 2 Instituto Nacional de Ciência e Tecnologia – Doenças Tropicais (INCT-DT), Brazil; 3 Centro de Pesquisa Gonçalo Moniz (Fiocruz), Salvador, Bahia, Brazil; 4 Departments of Internal Medicine and Microbiology, University of Iowa and the VA Medical Center, Iowa City, Iowa, United States of America; 5 Departamento de Ciências da Biointeração, Instituto de Ciências da Saúde, Universidade Federal da Bahia, Salvador, Bahia, Brazil; Philipps-Universitat Marburg, GERMANY

## Abstract

GP63 or leishmanolysin is the major surface protease of *Leishmania* spp. involved in parasite virulence and host cell interaction. As such, GP63 is a potential target of eventual vaccines against these protozoa. In the current study we evaluate the polymorphism of *gp63* in *Leishmania (Viannia) braziliensis* isolated from two sets of American tegumentary leishmaniasis (ATL) cases from Corte de Pedra, Brazil, including 35 cases diagnosed between 1994 and 2001 and 6 cases diagnosed between 2008 and 2011. Parasites were obtained from lesions by needle aspiration and cultivation. Genomic DNA was extracted, and 405 bp fragments, including sequences encoding the putative macrophage interacting sites, were amplified from *gp63* genes of all isolates. DNA amplicons were cloned into plasmid vectors and ten clones per *L*. *(V*.*) braziliensis* isolate were sequenced. Alignment of cloned sequences showed extensive polymorphism among *gp63* genes within, and between parasite isolates. Overall, 45 different polymorphic alleles were detected in all samples, which could be segregated into two clusters. Cluster one included 25, and cluster two included 20 such genotypes. The predicted peptides showed overall conservation below 50%. In marked contrast, the conservation at segments with putative functional domains approached 90% (Fisher’s exact test p<0.0001). These findings show that *gp63* is very polymorphic even among parasites from a same endemic focus, but the functional domains interacting with the mammalian host environment are conserved.

## Introduction

The *Leishmania* species parasite cause a variety of clinical syndromes in individuals living in tropical and subtropical areas of the globe [1]. An estimated 1.5 to 2 million people develop symptomatic leishmaniasis per year, and approximately 12 million people are infected worldwide [2]. American Tegumentary Leishmaniasis (ATL) refers to cutaneous forms of leishmaniasis in the New World, caused by species of the subgenera *Leishmania Viannia* and *Leishmania Leishmania* [1, 3]. *L*. *(V*.*) braziliensis* is the main cause of ATL in South America, including Brazil [4, 5].

The standard treatment for ATL in Brazil is pentavalent antimony [Sb(V)] at a daily dose of 15–20 mg/Kg for 20 to 30 days [5]. However, up to 50% of patients fail Sb(V) therapy [6–9]. This scenario is even worse in areas of high *L*. *(V*.*) braziliensis* endemicity such as Corte de Pedra in the state of Bahia, Brazil. The most common form of disease due to *L*. *(V*.*) braziliensis*, and the most responsive to therapy, is cutaneous leishmaniasis (CL). More difficult to treat forms of ATL in the region include the classically recognized mucosal leishmaniasis (ML), and emerging clinical forms such as disseminated leishmaniasis (DL) and atypical cutaneous leishmaniasis (ACL). Patients with these unusual forms tend to respond poorly to Sb(V), with failure occurring in up to 60–90% of these individuals [10–16]. Such experience has led investigators to realize the need for new treatment modalities for ATL [9, 16, 17].

Leishmanolysin, or glycoprotein 63 (GP63), is a major surface protease (MSP) of *Leishmania* [18–21], capable of hydrolyzing a variety of substrates in the parasite’s immediate environment within the host [18]. GP63 genes are expressed in promastigotes and amastigotes, and their products are involved in the adhesion to and internalization of the parasite by the host macrophages [19, 22–25]. Furthermore, GP63 is in part responsible for *Leishmania* spp. to migrate through extracellular matrix, to avoid lysis by inactivating components of the complement system, and to hydrolyze intracellular macrophage targets [26, 27]. Its increased expression has been correlated with increased virulence of *L*. *(V*.*) braziliensis* [28].

Given its role in pathogenesis, GP63 might prove a good target for treatment of or prophylactic immunization against leishmaniasis [21, 29]. Its use might be complicated by the variability in genes encoding these molecules [30, 31]. However, peptides synthesized according to short regions in GP63, conserved across different species of *Leishmania*, have been shown to inhibit internalization of the parasite by macrophages [32]. Effective inhibitory oligopeptides surround the sequences SRYD, involved in binding macrophage surface receptors, and HExxH, an essential region for metalloprotease activity [32]. The goal of the current study was to determine whether these short regions are conserved in *gp63* genes from a panel of *L*. *(V*.*) braziliensis* isolates from a variety of patients whose diagnosis is temporally distributed over time. The study utilized parasites isolated from ATL patients of Corte de Pedra, Brazil. The results provide the basis for considering these peptides for therapeutic use in management of leishmaniasis. Furthermore, polymorphic *gp63* alleles could potentially serve as molecular markers of functionally distinct *L*. *(V*.*) braziliensis* isolates.

## Materials and Methods

### Study area

Corte de Pedra is composed of 20 municipalities in a rural area located in the southeastern region of the state of Bahia, in the northeast of Brazil within geographic coordinates (latitude/longitude) 14°/39°, 13°/39°, 14°/40°, 13°/40°. *Lutzomyia (Nyssomyia) whitmany* and *L*. *(N*.*) intermedia* sandflies are the most important vectors of *L*. *(V*.*) braziliensis* in this endemic area [33]. The residents work mostly in agriculture, a vocation that often takes them into primary or secondary forests. There is little population migration in or out of this region. Study participants’ mean time of residence at their addresses at the time of diagnosis and parasite sampling was 17 years. 90% of the study participants lived on farms.

### ATL patients’ disease definitions

CL consists of an ulcerated skin lesion at a single body site with no more than two secondary or satellite lesions, without clinical evidence of mucosal involvement. ML was defined as the presence of an inflamed or ulcerated mucosal lesion(s) at a site that is noncontiguous with any cutaneous lesion. Subjects with ML might have concomitant lesions of CL, but not always. DL was defined as 10 or more skin lesions of mixed types (acneiform, papular, nodular, and/or ulcerated) located on two or more body parts (head, trunk, arms and legs).

### Parasites

The *L*. *(V*.*) braziliensis*is isolates analyzed in the present study were obtained by culture of aspirates from the borders of skin or mucosal lesions. Aspirate material was immediately suspended in biphasic liver infusion tryptose-Novy, McNeal and Nicolle medium (LIT/NNN) and incubated at 26°C for 1 to 2 weeks. The suspension was transferred to Schneider’s medium complemented with 10% heat-inactivated fetal calf serum and 2 mM L-glutamine, and incubated at 26°C for up to an additional 2 weeks. Parasites were frozen without further subculture in 10% dimethyl sulfoxide (DMSO) 90% growth medium and maintained in liquid nitrogen until used.

Thirty-five *L*. *(V*.*) braziliensis* isolates were obtained from 17 individuals with CL, 9 with ML and 9 with DL, between 1992 and 2001. Six *L*. *(V*.*) braziliensis* isolates derived from 2 individuals with CL, 2 with ML and 2 with DL diagnosed between 2008 and 2011. All patients were diagnosed at the outpatient clinic in the health post of Corte de Pedra.

### *L*. *(V*.*) braziliensis* genomic DNA extraction and parasite species determination by PCR

Genomic DNA was extracted from approximately 10^6^ promastigotes of each isolate. Briefly, parasites were pelleted and suspended in 150μL of TELT buffer (50mM Tris-HCl pH 8.0, 62.5mM EDTA pH 9.0, 2.5M LiCl, 4% v/v Triton x 100) for 5 min at room temperature, followed by phenol-chloroform extraction (150μL) to remove protein and lipids. Nucleic acids were precipitated with ethanol (300μL), followed by an ethanol rinse (1,000μL). The pellets were suspended in 100μL of TE buffer (Tris-HCl 10mM, EDTA 1mM pH 8.0). Samples were stored at either –20°C or -70°C until used. The determination of the infecting *Leishmania* species was performed by real-time qPCR assay, using primers based on sequences of KDNA1, KDNA3 and MAG1 as previously described [34].

### PCR amplification and cloning of selected targets

The *gp63* used as reference for the alignment encodes a 557 amino acids peptide: "*Leishmania braziliensis* MHOM/BR/75/M2904 GP63 leishmanolysin (LBRM_10_0540)", accessed at www.ncbi.nlm.nih.gov/nuccore/XM_001562773.2. We focused the regions that encode the putative functional sites (macrophage binding, protease activity) of GP63 within the host [32]. A fragment of *gp63* genes containing these sites was amplified with the forward 5:ATGTCCCGCGACCGCAGCAG and reverse 5:TCACACCGCCGCTGTGTCGG primers in a 50μL reaction volume, in a 96-well thermal cycler Veriti^®^ from Applied Biosystems.

Amplified products were separated in 1.3% agarose gels, stained with ethidium bromide and visualized with a UV trans-illuminator—digital imager (UVP Labworks Laboratory Imaging and Analysis System Inc., CA, EUA) in order to confirm the amplicons were the expected 405 bp size.

Amplified *gp63* fragments were cloned using the Original TA Cloning Kit pCR 2.1 VECTOR (Invitrogen), according to manufacturer’s instructions. The recombinant PCR 2.1 plasmids were transformed into competent DH5α *Escherichia coli* [35]. Ten colonies with inserts were selected per studied *L*. *(V*.*) braziliensis* isolate.

### Sequence analysis

Plasmid inserts were sequenced by the Sanger method at Macrogen Inc. (Seoul, South Korea), with M13 sequencing primers flanking PCR 2.1 cloning sites. Sequencing was bidirectional and only sequences with 100% identity among overlapping insert strands were recorded. Insert sequences were aligned to the *Leishmania braziliensis* MHOM/BR/75/M2904 GP63 leishmanolysin (LBRM_10_0540) gene sequence with MEGA 5.0 software. ClustaW algorithm was used in the process and no manual adjustments to maximize sequence alignments were necessary.

Upon alignment we searched for events of SNP and indels among the cloned 405 bp *gp63* fragments. Polymorphism among sequences of *gp63* fragments and their predicted peptides was evaluated manually and confirmed using the Dna Sequence Polymorphism software, version 5.10.01 [36, 37]. We defined a polymorphism as a single bp difference between isolates; a polymorphic allele as a linear *gp63* DNA sequence detected in more than one clone per parasite isolate, and in more than one isolate of *L*. *(V*.*) braziliensis* in our study sample.

Classification of *gp63* alleles employed Neighbor Joining algorithm. Consistency of nodes within dendrogram was tested by bootstrap.

### Statistical Analysis

Differences in the distribution frequencies of each polymorphism detected among *gp63* fragments were analyzed by Fisher’s exact test. Results with p ≤ 0.05 were considered statistically significant.

### Ethics statement

This study was approved by the institutional review board of the Federal University of Bahia Medical College, under document number CAAE: 37297614.0.0000.5577.

## Results

### Identification of polymorphisms in the vicinity of the putative macrophage binding region of *gp63* among human isolates of *L*. *(V*.*) braziliensis*

**Fig 1:**
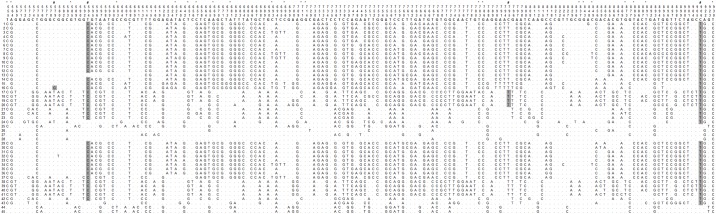
Alignment of polymorphic nucleotide positions of 45 polymorphic alleles found for the *gp63* gene fragment studied. Alleles 1 to 28 were detected among 35 different isolates of *L*. *(V*.*) braziliensis* from ATL patients diagnosed between 1992 and 2001 in Corte de Pedra, Brazil. Alleles 29 to 45 were detected among isolates of the parasite drawn from six different patients of the same area between 2008 and 2011. Alignment was generated by MEGA 5.0 software. Numbers boxed in top three rows correspond to nucleotide position in the *gp63* gene used as reference (see methods). Top sequence in bold corresponds to the nucleotides found in each displayed position in the reference *gp63* sequence. Rows numbered 1 to 45 at left correspond to the 45 alleles of *gp63* detected in Corte de Pedra. Dots indicate same nucleotide as reference sequence, while letters indicate the substituting nucleotides in the study fragments. (*) Positions with silent polymorphisms in study *gp63* fragments. (#) Positions with polymorphisms that may be silent or lead to predicted amino acid substitution (highlighted in gray), depending on the study *gp63* allele. All other positions resulted in predicted amino acid substitution in study *gp63* alleles.

Forty-five alleles of *gp63* could be distinguished among 410 clones of the gene fragment evaluated in thirty-five isolates of *L*. *(V*.*) braziliensis* collected between 1992 and 2001, and six isolates of the parasite drawn between 2008 and 2011 from ATL patients of Corte de Pedra. The median number of alleles per parasite isolate was 5, ranging from 2 to 9. One hundred eighty-six polymorphic positions were found in the 405 bp fragment, considering all forty-five alleles of the gene (Fig 1). This results in an average of one polymorphic position every 2.2 base-pairs. The frequency of polymorphic positions varied from eight in allele 28 to one hundred one in allele 8 (Fig 1), with a median of 93 polymorphisms per allele (Table 1). 169 polymorphisms consisted of nucleotide substitutions. Many variations occurred in the first two nucleotides of codons, predicting amino acid replacements in 73 (54%) of the translated proteins. Silent changes were also observed in only 52 (31%) nucleotide positions. Nucleotide positions 579, 594, 795 and 906 had either amino acid replacement or silent polymorphisms in different *gp63* alleles (Fig 1).

**Table 1:** Counts of nucleotide/amino acid polymorphisms in nucleic acid/predicted amino acid sequences from 405 base-pairs study fragments of *L*. *(V*.*) braziliensis gp63*. Columns show data for all forty-five *gp63* alleles (total) identified and alleles stratified according to clustering analysis depicted in Fig 2 (clades A to D).

	TOTAL	CLADE A	CLADE B	CLADE C	CLADE D
	(nt/aa)	(nt/aa)	(nt/aa)	(nt/aa)	(nt/aa)
**Number of alleles**	45	25	8	5	7
**Lowest count of polymorphisms per allele**	8/7	86/39	89/47	50/29	8/7
**Highest count of polymorphisms per allele**	101/52	101/45	93/52	54/35	49/30
**Median count of polymorphisms per allele**	93/43	94/43	92,5/51	53/34	42/27

* nt/aa = nucleotides/amino acids.

### Classification of *gp63* alleles

**Fig 2:**
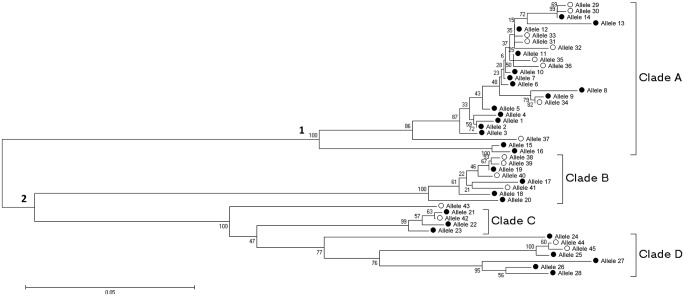
Neighbor-Joining classification of 45 alleles of the *gp63* study fragment found in 41 isolates of *L*. *(V*.*) braziliensis* from Corte de Pedra, Brazil. Nucleotide sequence alignments and *gp63* allele classification employed MEGA 5.0 software. Branch tips correspond to each *gp63* fragment allele. Clusters 1 and 2 correspond to the major aggregates, while clades A to D correspond to the secondary aggregates of alleles. Black dots indicate alleles detected among 35 different isolates of *L*. *(V*.*) braziliensis* obtained from ATL patients in Corte de Pedra between 1992 and 2001. White dots indicate alleles in parasites drawn from six different patients of the same area between 2008 and 2011. Percentages at the nodes of the dendrogram consist in bootstrap values.

Neighbor-Joining classification revealed two distinct clusters of alleles, which could be further subdivided into four smaller clades (Fig 2; clades A to D). We defined as clade each discrete group of *gp63* alleles immediately lower hierarchically to the cluster nodes in the dendrogram. Cluster 1 included 25 *gp63* alleles, all belonging to clade A. Cluster 2 included 20 alleles distributed across clades B, C and D.

### The predicted peptides reveal that segments of *gp63* encoding functional peptides are highly conserved in the natural population of *L*. *(V*.*) braziliensis*

**Fig 3:**
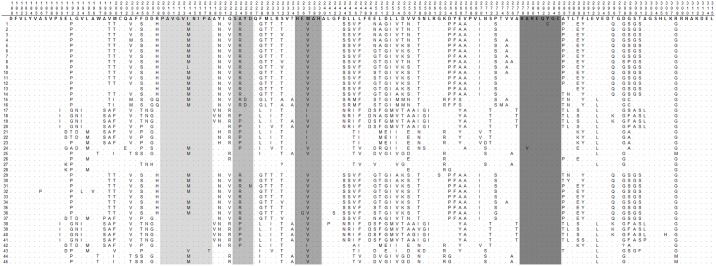
Alignment of amino acid positions among peptides predicted from 45 alleles found for the *gp63* gene fragment studied. The 45 translated alleles were detected among 41 different isolates of *L*. *(V*.*) braziliensis* from ATL patients of Corte de Pedra. Alleles 1 to 28 derived from 35 parasite isolates obtained from ATL patients between 1992 and 2001. Alleles 29 to 45 derived from 6 parasite isolates obtained from ATL patients between 2008 and 2011. Alignment was generated by MEGA 5.0 software. Numbers boxed in top three rows correspond to amino acid positions in the *gp63* gene used a reference (see methods). Top sequence in bold corresponds to the amino acids found for each displayed position in the reference *gp63* sequence. Rows numbered 1 to 45 at left correspond to the 45 alleles of *gp63* detected in Corte de Pedra. Dots indicate same amino acids as in reference sequence, while letters indicate the substituting amino acids in the study fragments. Regular letters indicate hydrophilic, italicized letters indicate intermediary, and bold letters indicate hydrophobic amino acids. Shaded positions correspond to the segments PAVGVINIPA, SRYD, HEVAH and KAREQYGC referred to in text.

The predicted peptide encoded by the *gp63* fragment is 135 amino acids long. The number of polymorphic amino acid positions varied from seven in allele 28 to fifty-two in alleles 38 and 40, with a median of 43 amino acid changes per allele (Fig 3, Table 1). In 54% of these polymorphic positions there were changes in classes of predicted amino acids between alleles (Fig 3). The 45 *gp63* translated alleles presented an overall conservation of 46%.

Despite the overall low conservation of the predicted GP63 fragment studied, the two previously described functional regions, defined by the primary sequences SRYD (macrophage receptor binding) and HEVAH (metalloprotease activity) [32] exhibited approximately 70% identity across the 45 *gp63* alleles. There were two other highly conserved short regions that flanked the SRYD and HEVAH sequences (PAVGNIPA and KAREQYGC). Altogether, these four stretches of amino acids encompassed 27 of the 135 evaluated residues (Fig 3, shaded positions). The data showed approximately 89% identity between these peptides across the predicted polypeptides of all *gp63* alleles sequenced. This is in marked contrast with the overall identity of 46% of predicted protein residues in the full peptide fragments (Fisher’s exact p <0.0001; Fig 3).

A BLAST search of Genbank deposited sequences using PAVGNIPA, HEVAH, SRYD and KAREQYGC further displayed that these segments were also conserved among *L*. *(V*.*) braziliensis* [38], *L*. *(V*.*) guyanensis* [39], *L*. *(V*.*) panamensis* [40], *L*. *(L*.*) Mexicana* [38], *L*. *major* [41], *L*.*donovani* [42] and *L*. *infantum* [38].

### Novel *gp63* alleles arise in natural *L*. *(V*.*) braziliensis* populations overtime

Twenty-eight alleles of *gp63* could be distinguished among 350 clones of the gene fragment evaluated in thirty-five isolates of *L*. *(V*.*) braziliensis*, collected between 1992 and 2001 from ATL patients of Corte de Pedra. The median number of alleles per parasite isolate was 5, ranging from 2 to 9. In the sample of parasites obtained from six ATL patients of Corte de Pedra between 2008 and 2011, twenty-five alleles could be discriminated among 60 clones of the 405 bp *gp63* fragment evaluated. The median number of alleles per parasite isolate was 4.5, ranging from 1 to 8. Remarkably, seventeen novel alleles could be identified in this latter sample (Fig 1, *gp63* 29 to 45). These alleles distributed throughout all defined clades of *gp63* in Corte de Pedra (Fig 2, white dots).

## Discussion

Polymorphic alleles of mammalian hosts have been a focus of investigations into the diverse outcome of infection with different *Leishmania* species. Our prior work has underscored the fact that polymorphic isolates of *L*. *(V*.*) braziliensis* are independently associated with different clinical outcomes of infection [43, 44]. These analyses have focused on anonymous markers that are not associated with any functional significance, in part due to a lack of knowledge of genomic markers in genes of functional significance. In the current study, we investigated the variability in the coding regions of *gp63* genes, focusing on peptides with known functional significance in the host. We were able to analyze the variability between *gp63* genes among clinical isolates from a population of individuals naturally infected with *L*. *(V*.*) braziliensis*. We found that *gp63* genes are highly polymorphic, but that sequences encoding the functional peptides involved in macrophage binding, or in protease activity were remarkably conserved among parasites from one of the regions with highest endemicity of ATL, in northeast Brazil.

GP63 is encoded by tandemly repeated gene clusters in all species in which the gene organization has been investigated [28]. Study of *gp63* gene cluster in *L*. *(V*.*) braziliensis* revealed approximately 37 genes in the cluster, with 8 distinct classes [31]. Our experimental approach, in which we selected 10 clones from each isolate for sequence analysis, could not distinguish the full spectrum of polymorphic *gp63* genes in single isolates. Nonetheless the polymorphism of the total population was much higher than expected from a single parasite clone, and the number of polymorphic alleles greatly exceeded the expected 8 reported. As such, this analysis leads to a reasonable conclusion that the polymorphism of *gp63* genes in the entire population exceeds that expected in individual *L*. *(V*.*) braziliensis* isolates. Further documentation of isolates containing different *gp63* genes will require the generation of allele-specific markers from our sequence information, and re-examination of these specific polymorphisms in the collection of clinical isolates.

Polymorphism concentrated in stretches of the gene that encode segments of GP63 that do not directly participate in the interaction between parasite and host cell. We speculate that population-wise the extensive variability of GP63 may be in part driven by selective pressure caused by the host immune responses, during infection with *Leishmania* spp. Studies suggest that GP63 polymorphisms are more abundant in segments of the protein that serve as epitopes for T and B cells [45, 46]. The existence of several dozen, and potentially hundreds of *gp63* alleles within a single *L*. *(V*.*) braziliensis* population might perhaps allow the parasite overcome host herd immunity, and maintain endemicity at each successive transmission season of ATL.

We detected four different clades of alleles, and a median of approximately five distinct alleles per parasite isolate. This suggests that *gp63* polymorphism in the natural population of *L*. *(V*.*) braziliensis* from Corte de Pedra may be warranted by multiple loci and / or gene copies distributed in different chromosomes. This hypothesis is consistent with observations in previous studies of both physical mapping and whole genome sequencing of *L*. *(V*.*) braziliensis* strains [47, 48]. It is important to note that we analyzed *gp63* amplified from the genomes of non-cloned parasites by specific PCR primers. As such, the findings reported herein may consist in an underestimation of the actual complexity of these loci, in as much as they may also be influenced by isolates comprised of multiple strains of *L*. *(V*.*) braziliensis*. Future studies should employ deep sequencing approaches to address these limitations. Besides, since we did not evaluate gene expression then we cannot conclude on the functionality of detected alleles.

GP63 and the complement receptor CR3 on host-cells seem to interact in part via the leishmanolysin segment containing SRYD amino acids [49]. SRYD is highly conserved among *Leishmania* spp., and monoclonal antibodies against this oligo-peptide inhibit internalization of *L*. *infantum* by macropages [32]. Four short stretches of the putative macrophage interacting segment of GP63 proved highly conserved among *L*. *(V*.*) braziliensis* from Corte de Pedra. These include SRYD and HExxH. The active site at the N-terminal proteinase domain of leishmanolysin contains the HExxH sequence conserved in all species of *Leishmania* [32].

The other two conserved amino acid stretches were PAVGNIPA and KAREQYGC, which flank SRYD and HExxH. The consistent conservation of PAVGNIPA and KAREQYGC among parasites of Corte de Pedra suggests that these segments may also play a role in *L*. *(V*.*) braziliensis* host cell interaction. Reinforcing this hypothesis, the review of sequences for other species of *Leishmania*, deposited in Genbank or published in different papers, reveal the presence for PAVGNIPA and KAREQYGC in all of them [[32], GeneBank accession numbers: CBZ24358.1, AIN96110.1, AAC39120.1, XP_001463700.1, AAA29240.1,XP_001562820.1 and AAA53688.1]. Nevertheless biological testing with inhibition assays is necessary to ascertain that these segments are really functional for the interplay between host and parasite cells.

Proteases fulfill important roles during host infection, microbe survival and pathogenicity in several protozoa [50, 51]. GP63 (also called leishmanolysin or MSP) is one such metallo-enzyme produced by *Leishmania* spp. [52–54]. GP63 has been described to participate in complement resistance, migration through extracellular matrix, establishment of intracellular parasitism and interference with intracellular microbicidal mechanisms of infected cells [55–59]. Finally, several studies in experimental cutaneous and visceral leishmaniasis (VL) have shown efficacy of various formulations of GP63 administered through different routes in protective immunity in mouse models [21, 60–68].

Experimental studies do not take into account the complexity of natural pathogen populations. As in the reports cited above, immunization and challenge are usually carried out with a single pathogenic strain and its components. This approach is sound, but does not take into account the variability that some antigens used as immunogens may present within and / or between human disease transmission foci.

As we demonstrate in this molecular epidemiology study, GP63 is one such example of a highly variable molecule. Thus experimental results may not easily translate into its successful use as an immune prophylaxis reagent in affected regions. Its ample variability reported for Corte de Pedra likely reflects the realm found in other affected regions as well.

The polymorphisms in *gp63* genes reported herein might prove particularly interesting as molecular markers of different parasite isolates. As we previously showed, distinct *L*. *(V*.*) braziliensis* clades are associated with different clinical outcomes of infection [43, 44]. If future investigations are able to discover that polymorphisms in this highly important functional protein associate with distinct outcomes of infection, this could have implications for GP63 function in different disease forms. Focusing on polymorphic markers avoids the need to define all *gp63* genes present in isolates, but rather highlights only those genes that are different between isolates. This approach might be expanded to study polymorphisms in other known *Leishmania spp*. proteins important in pathogenesis.
